# Enhancing tuberculosis vaccine efficacy with a heterologous mRNA-ChAdOx1 prime-pull strategy targeting lung-resident memory T cells

**DOI:** 10.3389/fimmu.2026.1807190

**Published:** 2026-04-20

**Authors:** Marcellus Korompis, Christopher J. De Voss, Shuailin Li, Alberta Ateere, Helen McShane, Elena Stylianou

**Affiliations:** The Jenner Institute, Nuffield Department of Medicine, University of Oxford, Oxford, United Kingdom

**Keywords:** ChAdOx1, heterologous vaccination, lung-resident memory T cells, mRNA vaccine, PPE15, prime-pull, tuberculosis

## Abstract

**Introduction:**

Tuberculosis (TB) remains a leading cause of morbidity and mortality worldwide, and Bacillus Calmette–Guérin (BCG) offers inconsistent protection against adult pulmonary TB. We previously showed that homologous mRNA vaccination encoding the mycobacterial antigen PPE15 (mRNA.PPE15) enhanced immunogenicity but did not improve protection over BCG alone. We hypothesised that a heterologous “prime-pull” strategy, systemic mRNA priming followed by mucosal adenoviral boosting, would enrich lung-resident memory T cells (TRM) and improve efficacy.

**Methods:**

Female C57BL/6 mice received BCG prime followed by subunit regimens combining intramuscular mRNA.PPE15 and intranasal ChAdOx1.PPE15 in different administration orders, alongside homologous controls. Cellular responses in spleen and lung were quantified by intracellular cytokine staining after PPE15 peptides stimulation. Intravascular staining was used to distinguish parenchymal (IV-) from vascular (IV+) cells and combined with tetramer staining to identify PPE15-specific CD4+ and CD8+ TRM-phenotype in the lung parenchymal following vaccination. PPE15-specific serum antibodies were measured by ELISA. Protective efficacy was assessed four weeks after aerosol *Mycobacterium tuberculosis* (*M.tb*) challenge by lung and spleen CFU enumeration.

**Results:**

Heterologous vaccination induced robust spleen CD4+ and CD8+ responses and PPE15-specific IgG. In the lung, mRNA.PPE15-ChAdOx1.PPE15 induced IFN-γ+ CD4+ and CD8+ T cells in the parenchyma and PPE15-specific TRM-like cells. Following *M.tb* challenge, both heterologous regimens reduced lung CFU compared to naïve controls, but only mRNA.PPE15-ChAdOx1.PPE15 significantly decreased CFU in both lungs and spleen. When used to boost BCG, BCG-mRNA.PPE15-ChAdOx1.PPE15 achieved 0.8 log10 CFU reductions in lungs and spleen compared to BCG control group and induced the greatest numbers of lung TRM-phenotype cells.

**Discussion:**

A heterologous prime-pull strategy that combines intramuscular mRNA.PPE15 priming with intranasal ChAdOx1.PPE15 boosting effectively directs PPE15-specific T cells to the lung parenchyma, enriches TRM-like populations, and improves protection over homologous regimens. There was a trend for the mRNA.PPE15-ChAdOx1.PPE15 regimen to outperform the reverse order, particularly as a BCG booster. These data support heterologous platform vaccination and prime-pull strategy as a novel strategy for TB vaccines and indicate potential to progress to the next stages of vaccine development.

## 1 Introduction

Tuberculosis (TB), caused by *Mycobacterium tuberculosis* (*M.tb*), remains a global health crisis, with an estimated 10.7 million cases and 1.23 million deaths reported in 2024 (1). The *Bacillus Calmette-Guérin* (BCG) vaccine, the only licensed TB vaccine (2), provides limited protection against pulmonary TB in adults (3). This has spurred efforts to develop vaccines to boost BCG efficacy (4). Among these, heterologous vaccination strategies, combining different vaccine platforms for priming and boosting, have shown promise by leveraging the unique strengths of each platform to induce robust and durable immune responses (5–10).

Despite advances, TB vaccine development remains hampered by the absence of defined immune correlates of protection. While immune parameters such as antigen-specific CD4+ T cells and IFN-γ are known to be important, they do not consistently correlate with vaccine efficacy (11, 12). This complicates the selection of antigen delivery platforms and necessitates empirical approaches. A range of platforms have been explored for TB vaccination, including viral vectors, protein-adjuvant combinations, and more recently mRNA-LNP technologies (5, 13–18). The latter gained momentum following their success in protecting against COVID-19 and have since attracted interest for TB vaccine immunisation strategies (19, 20). Although mRNA-based vaccines have shown strong immunogenicity and efficacy in preclinical studies, their ability to consistently improve BCG-mediated protection has been mixed (17, 18, 21). Recent preclinical studies report that selected multivalent mRNA-LNP constructs can augment or even exceed BCG protection, but outcomes appear to depend on antigen, formulation and delivery strategy (21).

We previously showed that an mRNA vaccine encoding PPE15, a mycobacterial antigen associated with the Esx-5a secretion system of *M.tb* (22–24), induced robust CD4+ and CD8+ Th1 responses, antibodies and improved BCG immunogenicity, but failed to protect against TB (17). We hypothesised that, while immunogenic, the mRNA induced PPE15-specific responses may not effectively localise to the lung, the primary site of *M.tb* infection. Prime-pull vaccination strategies, which combine systemic priming with mucosal boosting have shown promise in directing antigen-specific T cells to the lung mucosa (9, 25, 26).

In this study, we tested a heterologous “prime-pull” approach, where systemic priming with mRNA.PPE15 was followed by intranasal boosting with ChAdOx1.PPE15. We used intravascular staining in combination with PPE15-specific tetramers, to characterise T cell localisation and phenotype in the lung parenchyma (27). We identified PPE15-specific tissue-resident memory T cells (TRM), by the expression of CXCR3 or PD1 and absence of KLRG1 (KLRG1-), a phenotype previously associated with protection against *M.tb* (9, 28–30).

Our findings demonstrate that the BCG-mRNA.PPE15-ChAdOx1.PPE15 regimen offers significantly superior protection compared to homologous regimens, likely due to enhanced localisation of TRM-like cells in the lung. This approach provides a promising framework for the development of next-generation TB vaccines that more effectively induce mucosal immunity.

## 2 Methods

### 2.1 Mice and immunisations

Six- to eight-week-old female C57BL/6 mice were purchased from Envigo, UK. Animals were group housed in individually ventilated cages under specific pathogen-free conditions, with constant temperature and humidity. Mice were vaccinated with BCG-Pasteur at 3.5x10^5^ Colony Forming Unit (CFU) intradermally (i.d.) in the ears of mice (25 μl on each side). BCG Pasteur (ATCC 35734) was prepared in house in Middlebrook 7H9 broth (Becton Dickinson, UK), supplemented with 10% albumin dextrose catalase enrichment (Sigma-Aldrich) and 0.05% polysorbate 80 (Becton Dickinson, UK). ChAdOx1.PPE15 was generated as previously described (30), while the mRNA.PPE15 formulation and *in vivo* expression were prepared as described previously (17). Vaccinations with ChAdOx1.PPE15 were performed intranasally (i.n.) drop-by drop over both nostrils, with 1×10^8^ infectious units (ifu) in a 50 μL volume, diluted in endotoxin-free PBS (Merck Life Science, UK). mRNA.PPE15 vaccines were delivered intramuscularly to the right leg (50 μL), at 5 μg per dose. For homologous mRNA, 3 weeks were allowed between prime and boost. For heterologous regimens, 4 weeks were allowed between prime and boost vaccine. For BCG-boosting experiments, 10 weeks were allowed between BCG-prime and booster vaccination. All procedures were performed in accordance with the UK Animals (Scientific Procedures) Act 1986, under project licence number PP2165164, granted by the UK Home Office. Animal studies were approved by the Animal Welfare and Ethical Review Board (AWERB), University of Oxford and were in accordance with the Animal Research: Reporting of *in vivo* Experiments (ARRIVE) guidelines. Animals were randomly assigned to vaccination groups. No animals were excluded from analysis other than for pre-specified humane endpoints. Group sizes were chosen based on our previous experiments evaluating TB vaccine regimens and were calculated to provide at least 99% powered to detect an effect using a two-sided test at a 2.5% alpha error rate. All vaccinations were done under short-term inhalational anaesthesia using vaporised IsoFlo^®^. All animals were humanely sacrificed at the end of each experiment by cervical dislocation.

### 2.2 Immunogenicity

Lung and spleen cells were extracted from naïve and vaccinated mice. Splenocytes were obtained by mashing. Lungs were chopped into small pieces, and digested with DNase and collagenase (Sigma) as described previously (31). Suspensions from both organs were treated with ACK lysis buffer (150 mM NH_4_Cl, 10 mM KHCO_3_, 0.1 mM Na_2_EDTA, pH 7.2-7.4) and resuspended in DMEM (Sigma-Aldrich).

Flow cytometry: Spleen and lung cells were stimulated with media or with 2 μg/ml of PPE15 peptide pool, composed of 15-mer peptides overlapping by 11 amino acids, spanning the conserved immunodominant fist part of the sequence (1–221 out of 391 amino acids) (30) (Peptide Protein Research, UK) or with purified protein derivative *M.tb* (PPD-T) (10 μg/mL) (AJ Vaccines) for 2 hours, before addition of Golgi plug (BD Biosciences) for a further 4 hours at 37°C. Plates were placed at 4°C overnight and cells stained the following day. Cells were stained with live/dead fixable stain (ThermoFischer Scientific) for 10 mins, followed by α-CD16/32 α-CD45R, α-TCRβ, α-CD8 (eBioscience, UK), α-CD4 (Biolegend, UK). Cells were fixed and permeabilised with Cytofix/Cytoperm then stained intracellularly with α- IFN-γ, -TNF-α, -IL-2, -IL-17 (eBioscience, UK) (**Supplementary Figure 1**). For tetramer staining, 2x10^6^ cells were stained with I-A(b) PPE15_1–15_ phycoerythrin (NIH Tetramer Facility, Atlanta, GA, USA) or H-2D^b^ PPE15_192–200_ -allophycocyanin (ProImmune, Oxford,UK) at 4 °C for 30 min, washed with PBS and stained with α- KLRG1, -CXCR3, -CX3CR1 (BioLegend, UK), and -PD1 (eBioscience, UK) (**Supplementary Figure 2**). Samples were run on a LSR II or LSR Fortessa X-20 flow cytometer, and the data were analysed using FlowJo (TreeStar, Ashland, OR, USA).

Intravascular staining: Mice were injected via the lateral tail vein with 100 μl of α-CD45.2 fluorescein isothiocyanate (eBioscience, UK) at 25 μg/ml. Two minutes later, the lungs were collected and chopped into small pieces using sterile scissors. The lung tissues were digested (with collagenase and DNase) for 40 minutes at 37 °C, after which they were forced through a 70 μm-mesh-size filter. The cells were washed and red blood cells lysed with ACK lysis buffer before final wash and resuspension. In the figures where CD45.2-FITC antibody was used for intravascular staining, CD45- (CD45.2-FITC negative) indicates the parenchymal population and CD45+ (CD45.2-FITC positive) the vascular cell population.

### 2.3 Enzyme-linked immunosorbent assay

Serum was obtained from blood collected by cardiac puncture. Nunc maxisorp 96-well plates (ThermoFisher Scientific) were coated with 2 μg/ml of PPE15 protein in PBS and incubated overnight at 4°C. Plates were washed with 0.05% Tween20 (Sigma-Aldrich) in PBS and blocked with 2.5% BSA/PBS for 2 hours, followed by a 2-hour incubation with serially diluted serum samples prepared in PBS/Tween (PBS/T). Plates were washed and incubated with either alkaline phosphatase-conjugated anti-IgG (1/5000) (Sigma-Aldrich) or anti-IgG1 (1/1000) or anti-IgG2c (1/1000) (BioRad, CA, USA) diluted in PBS/T for one hour at room temperature. Development was performed with 1 mg/mL of 4-nitrophenylphosphate tablet (Sigma-Aldrich) diluted in diethanolamine buffer (ThermoFisher Scientific). Optical density (OD) was measured at 405 nm (Gen5 software) using a spectrophotometer (BioTeK Microplate Reader).

### 2.4 Mycobacterial challenge

Mice were placed in nose-only restrainers, and a Biaera Aero-MP-controlled nebuliser (Biaera Technologies, USA) was used to infect mice with aerosolised *M.tb* Erdman K01 (TMC107, BEI Resources, USA) at an airflow rate of 12 L/min and a pressure of 20 lb/in^2^ gauge, within a biosafety level 3 Total Containment Oxford Ltd isolator. Infection dose was confirmed 1-hour post-infection using two animals per run (10–40 CFU range). Lungs and spleen were collected 4 weeks after infection, homogenised in re-inforced tubes (Stretton Scientific) and a Precellys 24 homogeniser. Homogenates were serially diluted in PBS, plated on Modified 7H11 plates (Animal and Plant Health Agency, UK) and incubated at 37°C for 4 weeks. Limits of detection (LOD) for non-BCG-primed mice were, lung = 1,666 CFU, spleen = 166 CFU. For BCG-primed mice the LOD were, lung = 166 CFU, spleen = 16 CFU. Samples with zero colonies were assigned a value of LOD/2 for plotting and statistical comparisons. Investigators performing CFU plating and colony counting were blinded to group allocation.

### 2.5 Statistical analysis

FloJo v10.10 was used to analyse flow cytometry data, following a defined gating strategy. All graph and statistical analyses were performed using GraphPad Prism 10 software (GraphPad Software Inc.). To determine statistical significance, statistical analysis was conducted using Kruskal-Wallis one way ANOVA with Dunn’s test for multiple comparisons. Values of p ≤ 0.05 were considered statistically significant. *p ≤ 0.05, **p ≤ 0.01, ***p ≤ 0.001, ****p ≤ 0.0001.

## 3 Results

### 3.1 Heterologous vaccinations elicit robust systemic cellular immunity and antibody responses

**Figure 1:**
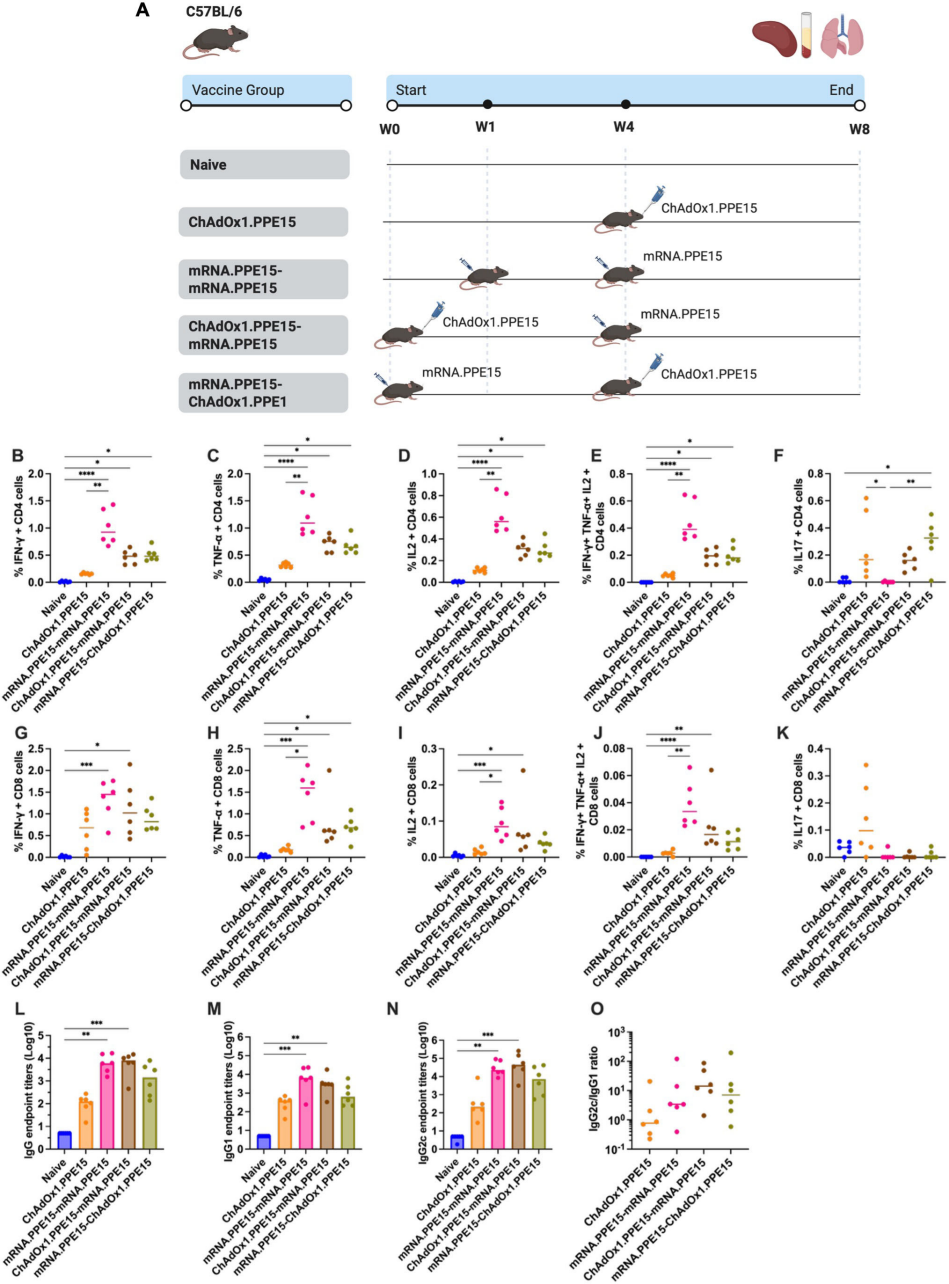
Robust splenic cellular and serum antibody responses following heterologous and homologous vaccination. **(A)** Experimental schema (created with BioRender.com). Immune responses were quantified four weeks after the last vaccination. Flow cytometric analysis of **(B)** IFN-γ, **(C)** TNF-α, **(D)** IL-2, or **(E)** all three of IFN-γ, TNF-α, and IL-2 and **(F)** IL-17 expression by CD4+ T cells after splenocytes were stimulated with immunodominant region of PPE15. **(G–K)** represent the same cytokines but for CD8+ T cells. **(L)** Serum PPE15-specific IgG, **(M)** IgG1, **(N)** IgG2c endpoint titres (starting dilution 1:10) and **(O)** the ratio of IgG2c to IgG1. Each symbol represents response from 1 animal, n=6 per group. The lines indicate the median value for each group. Statistical analyses were performed using Kruskal-Wallis, followed by Dunn’s multi-comparison test to evaluate differences between groups (all pairwise). *p<0.05, **p<0.01, ***p<0.001, ****p<0.0001.

To assess the immune responses across different vaccination regimens, mice were either left unvaccinated or received one of the following: a single intranasal dose of ChAdOx1.PPE15, a homologous intramuscular prime-boost with mRNA.PPE15-mRNA.PPE15 or a heterologous prime-boost combining intranasal ChAdOx1.PPE15 and intramuscular mRNA.PPE15, as shown in **Figure 1A**.

In the spleen, both the heterologous regimens and homologous mRNA.PPE15-mRNA.PPE15 induced significantly higher levels of CD4+ T cells producing IFN-γ, TNF-α, IL-2, as well as polyfunctional CD4+ T cells co-expressing all three cytokines, compared to naïve control ((N vs M-M (p<0.0001, <0.0001, <0.0001, <0.0001), N vs C-M (p=0.03, 0.02, 0.02, 0.03), N vs M-C (p=0.02, 0.03, 0.04, 0.02), **Figures 1B–E**). In contrast, intranasal ChAdOx1.PPE15 alone induced low but detectable CD4+ responses (**Figures 1B–E**). We also measured IL-17 responses since this cytokine has previously been associated with protection (32, 33). Notably, mRNA.PPE15-mRNA.PPE15 alone did not induce any IL-17 secreting CD4+ T cells (**Figure 1F**). However, the heterologous mRNA.PPE15-ChAdOx1.PPE15 regimen induced the highest level of CD4+ T cells producing IL-17, with detectable, but not significant responses also observed in the ChAdOx1.PPE15 and ChAdOx1.PPE15-mRNA.PPE15 groups (N vs C (p=0.057), N vs C-M (p=0.17), N vs M-C (p=0.01), **Figure 1F**).

A similar pattern was observed for CD8+ T cell responses. Both the mRNA.PPE15-mRNA.PPE15 and heterologous regimens induced higher frequencies of single (IFN-γ, TNF-α, IL-2) and polyfunctional CD8+ T cells compared to naïve control (N vs M-M (p= 0.0009, 0.0001, 0.0003, <0.0001), N vs C-M (p= 0.01, 0.03, 0.01, 0.0086), N vs M-C (p=0.06, 0.01, 0.07, 0.12), **Figures 1G–J**). Low and variable levels of IL-17 secreting CD8+ T cells were detected in the ChAdOx1.PPE15 group, whilst they were undetectable in all other groups (**Figure 1K**).

We next measured PPE15-specific antibody responses in serum at the end of the study. Significantly higher levels of IgG, IgG1 and IgG2c were detected in the mRNA.PPE15-mRNA.PPE15 and ChAdOx1.PPE15-mRNA.PPE15 groups compared to unvaccinated control (N vs M-M (p=0.0013, 0.0003, 0.0026), N vs C-M (p=0.0006, 0.0055, 0.0005)). Although antibody responses were also detected in the other vaccinated groups, they did not reach statistical significance (**Figures 1L–N**). Both the heterologous regimens and mRNA.PPE15-mRNA.PPE15 induced an IgG2c:IgG1 ratio greater than 1, indicating a Th1 biased response (**Figure 1O**).

### 3.2 Heterologous vaccinations elicit robust lung cellular immunity

Local antigen-specific responses at the site of *M.tb* infection are important for protection (28, 34, 35). We therefore also measured antigen-specific responses in the lung following vaccination.

**Figure 2:**
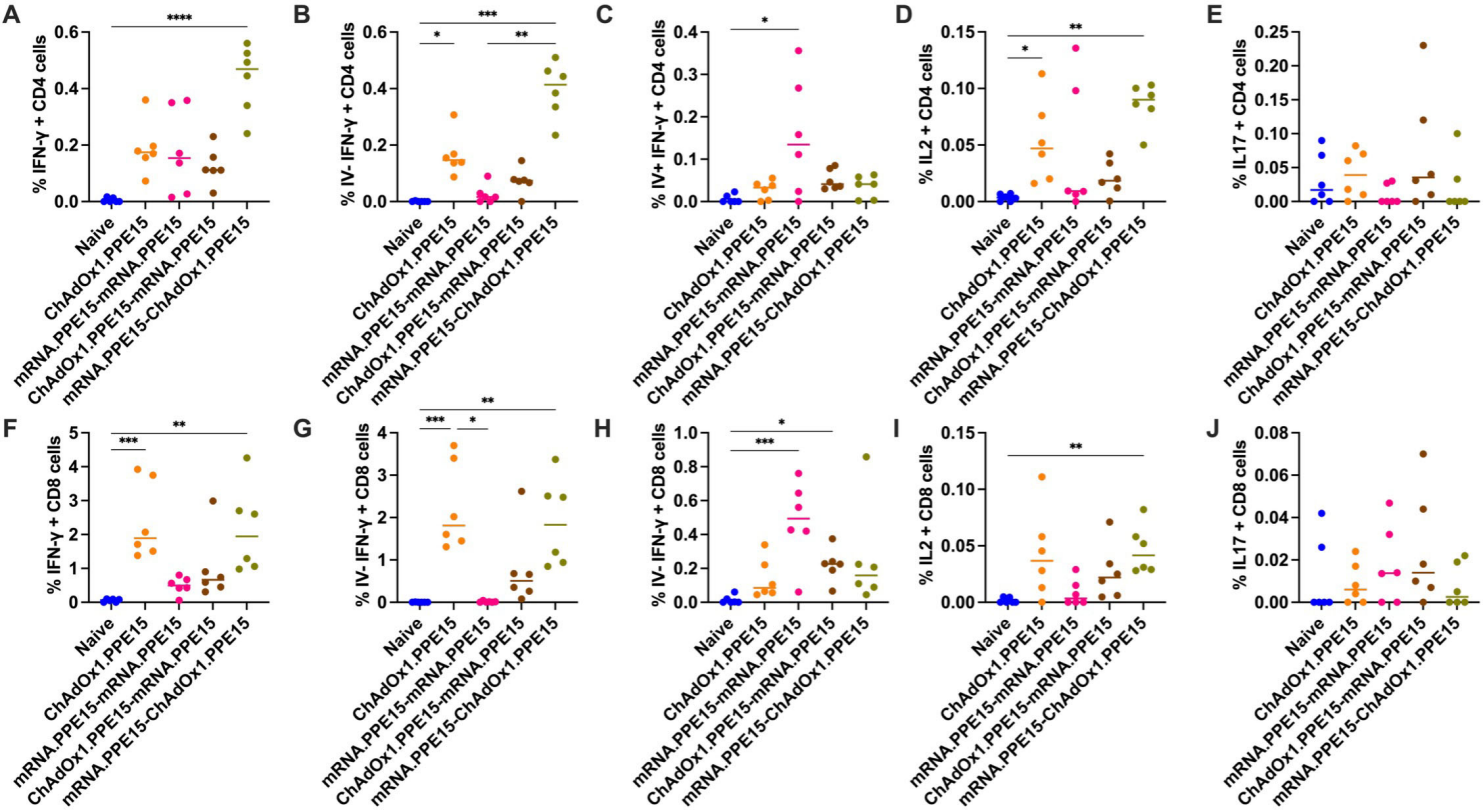
Robust PPE15-specific T cell responses in the lung following heterologous and homologous vaccination. Four weeks after the last vaccination lung cells were harvested following intravascular staining with αCD45 and stimulated with PPE15 peptide pool. Flow cytometric analysis of **(A)** IFN-γ, number of CD4+ T cells expressing IFN-γ, **(B)** intravascularly protected, **(C)** intravascularly stained, **(D)** IL-2, and **(E)** IL-17 expression by CD4+ T cells from lung cells stimulated with immunodominant region of PPE15. Panels **(F–J)** represent the same cytokines, but for CD8+ T cells. Each symbol represents response from 1 animal, n=6 per group. The lines indicate the median value for each group. Statistical analyses were performed using Kruskal-Wallis, followed by Dunn’s multi-comparison test to evaluate differences between groups (all pairwise). *p<0.05, **p<0.01, ***p<0.001, ****p<0.0001.

The mRNA.PPE15-ChAdOx1.PPE15 regimen induced the highest frequency of CD4+ T cell secreting IFN-γ compared to naïve control, while all other regimens elicited comparable IFN-γ responses (N vs M-C (p<0.0001), **Figure 2A**). We next conducted intravascular staining to distinguish lung parenchymal (IV-) from intravascular cells (IV+) (9, 30). The majority of IFN-γ responses in the ChAdOx1.PPE15 and mRNA.PPE15-ChAdOx1.PPE15 group were IV-, indicating localisation within the lung parenchyma (N vs C (p=0.02), N vs M-C (p=0.0002), **Figure 2B**). ChAdOx1.PPE15 induced a higher proportion of parenchymal responses compared to intravascular, while in the ChAdOx1.PPE15-mRNA.PPE15 group, responses were distributed more evenly across both compartments. In contrast, the majority of PPE15-specific CD4+ IFN-γ producing T cells were confined to the lung vasculature in mice vaccinated with mRNA.PPE15-mRNA.PPE15 (N vs M-M (p=0.01), **Figure 2C**). There is a trend for higher IV- CD4+ IFNγ and IL2 responses in the M-C compared to the C-M group (median difference 0.32 95% CI 0.19-0.43, p=0.07). Significant IL-2 responses were observed only in the ChAdOx1.PPE15 and mRNA.PPE15-ChAdOx1.PPE15 groups compared to naïve control (N vs C (p=0.04), N vs M-C (p=0.001), **Figure 2D**), while IL-17 responses were low across all vaccination regimens (**Figure 2E**).

CD8+ T cells producing IFN-γ were higher in frequency in the ChAdOx1.PPE15 and mRNA.PPE15-ChAdOx1.PPE15 groups compared to naïve control, with lower but detectable responses observed in the other vaccination regimens (N vs C (p=0.0005), N vs M-C (p=0.0019), **Figure 2F**). Similar to the CD4+ T cell profile, ChAdOx1.PPE15 induced a primarily parenchymal localisation of CD8+ T cells (N vs C (p=0.0006), **Figure 2G**). In the heterologous groups, CD8+ T cell IFN-γ responses in the ChAdOx1.PPE15-mRNA.PPE15 group were distributed across both compartments, whereas in the mRNA.PPE15-ChAdOx1.PPE15 group, the majority of responding cells resided in the lung parenchyma (N vs C-M (0.12, 0.02), N vs M-C (0.003, 0.12), **Figure 2G**). In contrast, CD8+ T cells in the mRNA.PPE15 group were predominantly localised in the vasculature (N vs M-M (p=0.0008), **Figure 2H**). Additionally, mRNA.PPE15-ChAdOx1.PPE15 induced significant IL-2 production in CD8+ T cells (N vs M-C (p=0.007), **Figure 2I**). CD8+ T cell IL-17 responses were low across all regimens (**Figure 2J**).

### 3.3 Heterologous vaccination enriches PPE15-specific lung-resident T cells phenotype in the parenchyma

Resident memory T cells (TRM) residing in the lung have been associated with protection in several TB studies (29, 30, 36). To further characterise PPE15 specific cells in the lung following vaccination, PPE15 I-A(b) and H-2D(b) tetramers (Tet) were combined with intravascular staining with an αCD45 antibody.

**Figure 3:**
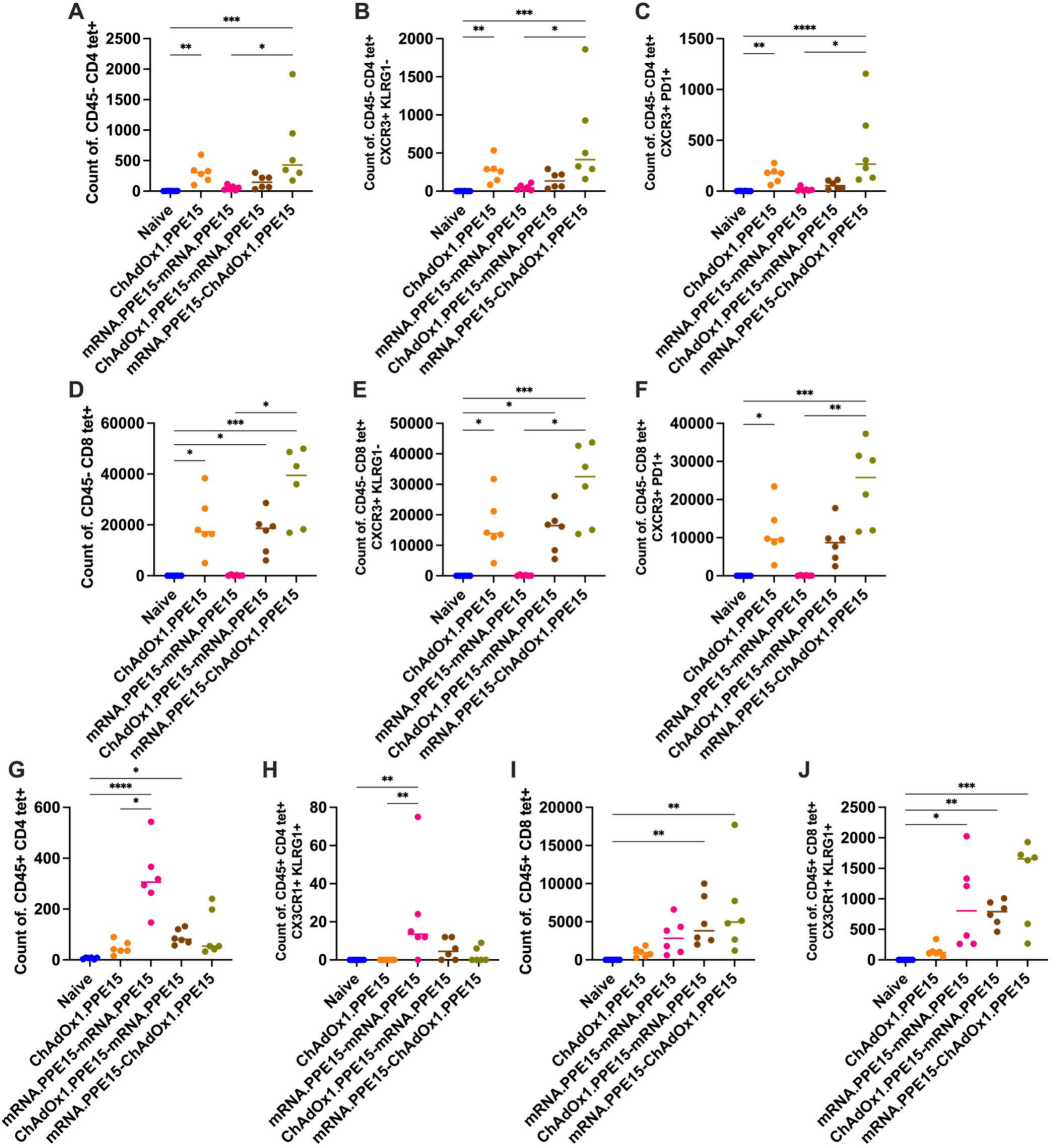
Phenotype characterisation of PPE15-specific T cells in the lung parenchyma and vasculature following heterologous vaccination. Four weeks after the last vaccination, PPE15 I-A(b) and H-2D(b) tetramers were combined with intravascular staining with αCD45. Number of tetramer-positive and intravascular staining-negative CD4+ T cells **(A)** that were **(B)** CXCR3+ and KLRG1- **(C)** CXCR3+ and PD1 +. Number of tetramer-positive and intravascular staining-negative CD8+ T cells **(D)** that were **(E)** CXCR3+ and KLRG1- **(F)** CXCR3+ and PD1 +. Number of tetramer-positive and intravascular staining-positive **(G, H)** CD4 + and **(I, J)** CD8 + T cells that were CX3CR1+ KLRG1 +. Each symbol represents response from 1 animal, n=6 per group. The lines indicate the median value for each group. Statistical analyses were performed using Kruskal-Wallis, followed by Dunn’s multi-comparison test to evaluate differences between groups (all pairwise). *p<0.05, **p<0.01, ***p<0.001, ****p<0.0001.

CD4Tet+ T cells were enriched in the lung parenchyma following vaccination with mRNA.PPE15-ChAdOx1.PPE15 and ChAdOx1.PPE15 alone (N vs M-C (p=0.0002), N vs C (p=0.003)). Although not significant compared to the naïve group, ChAdOx1.PPE15-mRNA.PPE15 also elicited CD4Tet+ T cells (N vs C-M (p=0.13)). mRNA.PPE15-mRNA.PPE15 induced the lowest number of CD4Tet+ T cells (**Figure 3A**). PPE15-specific cells in the parenchyma were mostly CXCR3+ KLRG1- (approximately 90%) and CXCR3+ PD1+, a phenotype characteristic of resident memory cells (29, 37) (**Figures 3B, C**). CD8+ T cells followed a similar trend, with ChAdOx1.PPE15, ChAdOx1.PPE15-mRNA.PPE15, and mRNA.PPE15-ChAdOx1.PPE15 having a significantly higher number of PPE15-specific cells compared to naïve control mice (N vs C (p=0.02), N vs C-M (p=0.02), N vs M-C (p=0.0003)). The mRNA.PPE15-ChAdOx1.PPE15 trended toward highest numbers of CD8Tet+ T cells (**Figure 3D**). Like CD4Tet+ T cells, most CD8Tet+ T cells were Tet+ CXCR3+ KLRG1- and Tet+ CXCR3+ PD1+ (**Figures 3E, F**). In contrast, within the lung vasculature, a different pattern emerged. The mRNA.PPE15-mRNA.PPE15 group exhibited the highest number of CD4Tet+ T cells, while responses in the other groups were generally lower, except for the ChAdOx1.PPE15-mRNA.PPE15 group, which also showed significantly elevated responses compared to naïve control (N vs M-M (p<0.0001,0.008), N vs C-M (p=0.02,0.45), **Figures 3G, H**). For CD8Tet+ T cells, a comparable number was detected in the mRNA.PPE15-mRNA.PPE15 and the heterologous groups, with the latter being significantly higher compared to the naïve control group (N vs M-M (p=0.06,0.01), N vs C-M (p=0.001,0.008), N vs M-C (p=0.001, 0.0007), **Figures 3I, J**). Low but detectable CD4+ and CD8+ T cell responses were also present in the vasculature of ChAdOx1.PPE15 vaccinated mice, with a proportion expressing CX3CR1 and KLRG1 (**Figures 3H, J**). These markers, associated with terminal differentiation, were previously shown to be absent in parenchymal cells but present in vascular T cells (9, 29).

### 3.4 Enhanced protection against *M.tb* in lungs and spleen following heterologous vaccination

**Figure 4:**
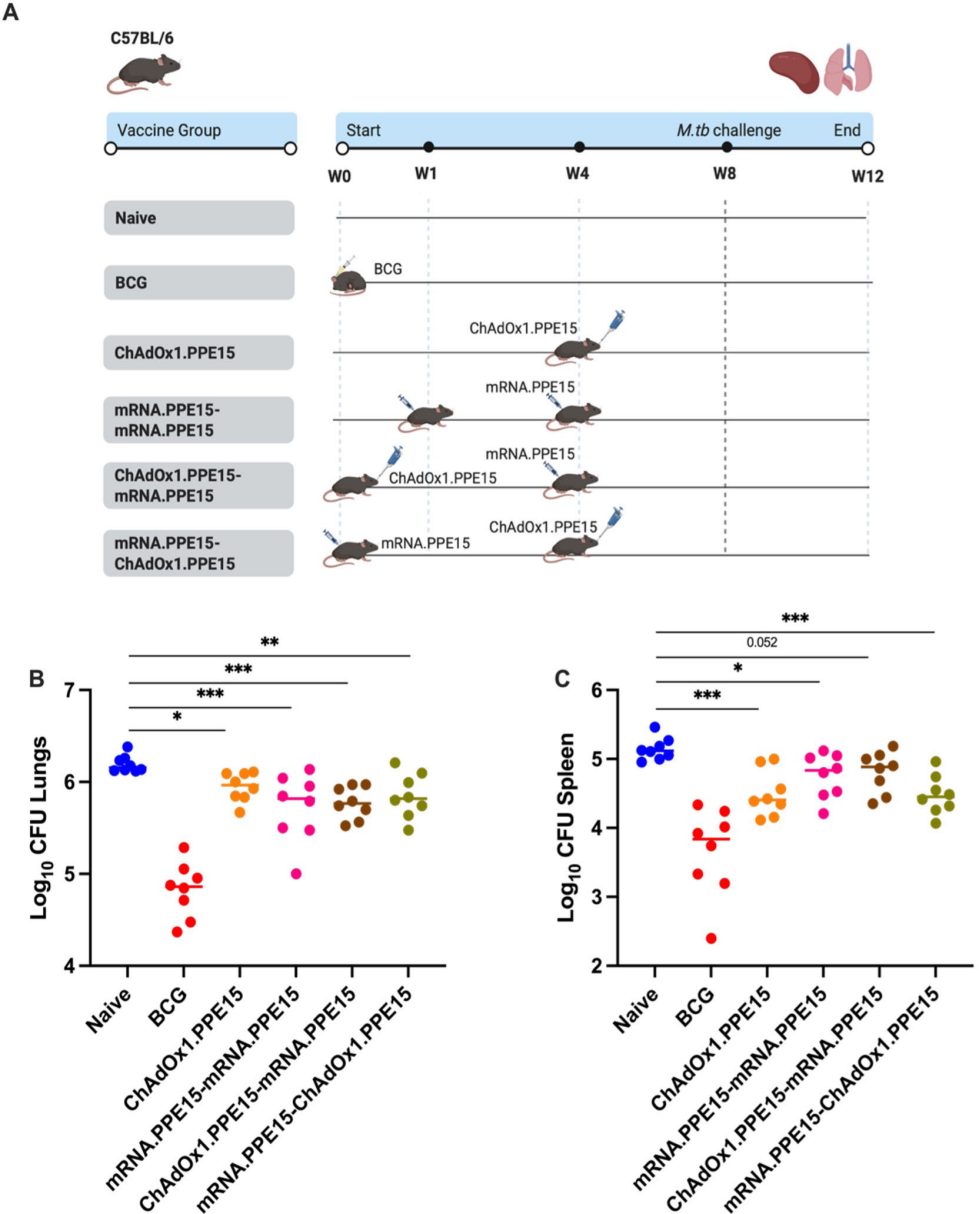
Protective efficacy of ChAdOx1.PPE15, mRNA.PPE15 and heterologous prime boost. **(A)** Experimental schema (created with BioRender.com). **(B)** Lungs and **(C)** spleens were harvested at the end of the study for colony-forming unit (CFU) enumeration. Each dot represents one animal, n=8 per group. The lines indicate the median CFU count. Statistical significance was determined using Kruskal-Wallis test with Dunn’s multiple comparisons test (all groups compared to naïve). *p ≤ 0.05, **p ≤ 0.01, ***p ≤ 0.001.

To determine, whether combining ChAdOx1.PPE15 with mRNA.PPE15, would improve the protective efficacy of the individual vaccines, mice were vaccinated as before, and subsequently challenged with aerosol *M.tb* (**Figure 4A**). Lung and spleens were collected four weeks after challenge for bacterial enumeration.

A small but significant reduction in the lung and spleen bacterial load was observed in the ChAdOx1.PPE15 and mRNA.PPE15-mRNA.PPE15 vaccinated groups compared to the naïve control (N vs C (p=0.016, 0.0003), N vs M-M (p=0.0004, 0.02)). Both heterologous vaccination regimens had significantly lower CFU in the lungs compared to the naïve control group (N vs C-M (p=0.0002), N vs M-C (p=0.0021), **Figure 4B**). However, in the heterologous groups, only the mRNA.PPE15-ChAdOx1.PPE15 group showed a significant reduction in spleen bacterial load (N vs C-M (p=0.052), N vs M-C (p=0.0002), **Figure 4C**). There was a trend toward lower spleen CFU in the mRNA.PPE15-ChAdOx1.PPE15 group compared with the ChAdOx1.PPE15-mRNA.PPE15 group however this did not reach statistical significance (M-C vs C-M spleen median difference -0.37log10 95%CI -0.64 to -0.02 p=0.11).

### 3.5 BCG-mRNA.PPE15-ChAdOx1.PPE15 induces robust systemic CD4+ and CD8+ T cell responses

**Figure 5:**
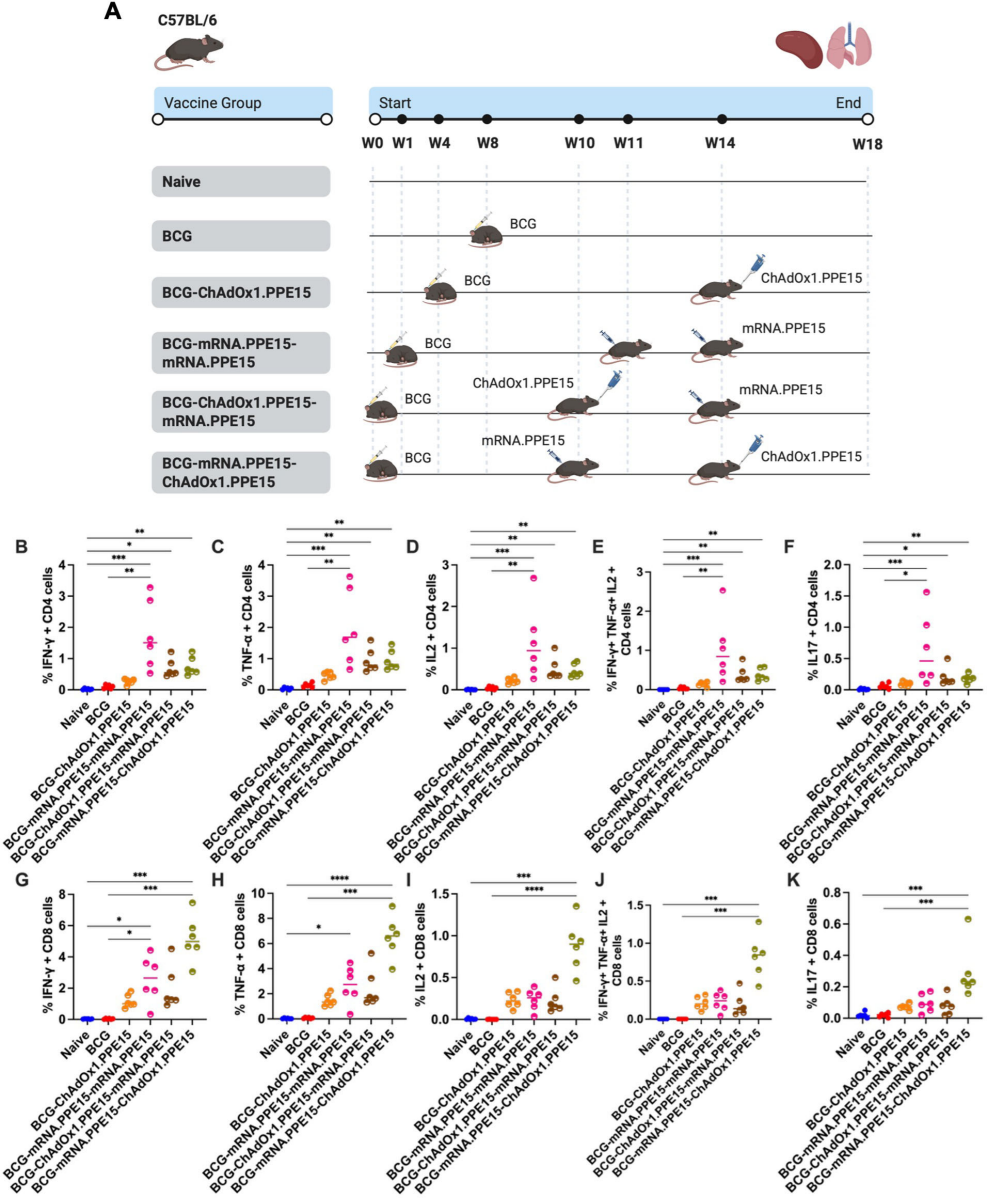
Spleen cellular and humoral responses following heterologous and homologous vaccination as a boost to BCG. **(A)** Experimental schema (created with BioRender.com). Immune responses were quantified four weeks after the last vaccination. Flow cytometric analysis of **(B)** IFN-γ, **(C)** TNF-α, **(D)** IL-2, **(E)** all three of IFN-γ, TNF-α, and IL-2 or **(F)** IL-17 expression by CD4+ T cells in the spleen, in response to stimulation with immunodominant region of PPE15. Panels **(G–K)**, represent the same cytokines, but for CD8+ T cells. Each symbol represents the response from 1 animal, n=6 per group. The lines indicate the median value for each group. Statistical analyses were performed using Kruskal-Wallis, followed by Dunn’s multi-comparison test to evaluate differences between groups (all pairwise). *p<0.05, **p<0.01, ***p<0.001, ****p<0.0001.

Subunit TB vaccines need to be effective at boosting BCG vaccination, typically administered during infancy as part of national immunisation programs. To determine whether these subunit vaccine regimens can improve the immunogenicity of BCG, mice were primed with BCG and boosted 10 weeks later with the different regimens (**Figure 5A**). Spleen and lungs were collected the end of study.

In the spleen, BCG-heterologous regimens and BCG-mRNA.PPE15-mRNA.PPE15 induced significant levels of CD4+ T cells expressing IFN-γ or TNF-α or IL-2 or triple-positive (IFN-γ+, TNF-α+, IL-2+) or IL-17 compared to naïve control (N vs B-C-M (p=0.01, 0.006, 0.004, 0.004, 0.012), N vs B-M-C (p=0.002, 0.002, 0.006, 0.002, 0.006), N vs B-M-M (p=0.0001, 0.0001, 0.0002, 0.0001, 0.0002). Vaccination with BCG-mRNA.PPE15 significantly improved PPE15-specific responses compared to BCG alone (B vs B-M-M (p=0.004, 0.005, 0.005, 0.009, 0.02), **Figures 5B–F**). BCG-ChAdOx1.PPE15 induced low CD4+ T cell responses.

For CD8+ T cells, BCG-mRNA.PPE15-ChAdOx1.PPE15 induced the highest frequency of cells expressing IFN-γ or TNF-α or IL-2, or triple-positive cells or IL-17, which was significantly higher compared to BCG and naïve controls (N vs B-M-C (p=0.0002, <0.0001, 0.0004, 0.0001, 0.0002, **Figures 5G–K**). CD8+ T cell responses were also detectable in all other vaccinated groups except for BCG.

To determine whether the immune activation by BCG was comparable across groups, responses following stimulation with PPD-T were measured. In the spleen, all BCG vaccinated groups had comparable IFN-γ, TNF, IL-2 and IL-17 production by CD4+ T cells following PPD-T stimulation (**Supplementary Figures 3A-D**). There was a non-significant induction of CD8+ T cells (**Supplementary Figures 3E-H**).

### 3.6 BCG-mRNA.PPE15-ChAdOx1.PPE15 induces robust lung CD4+ and CD8+ T cell responses

**Figure 6:**
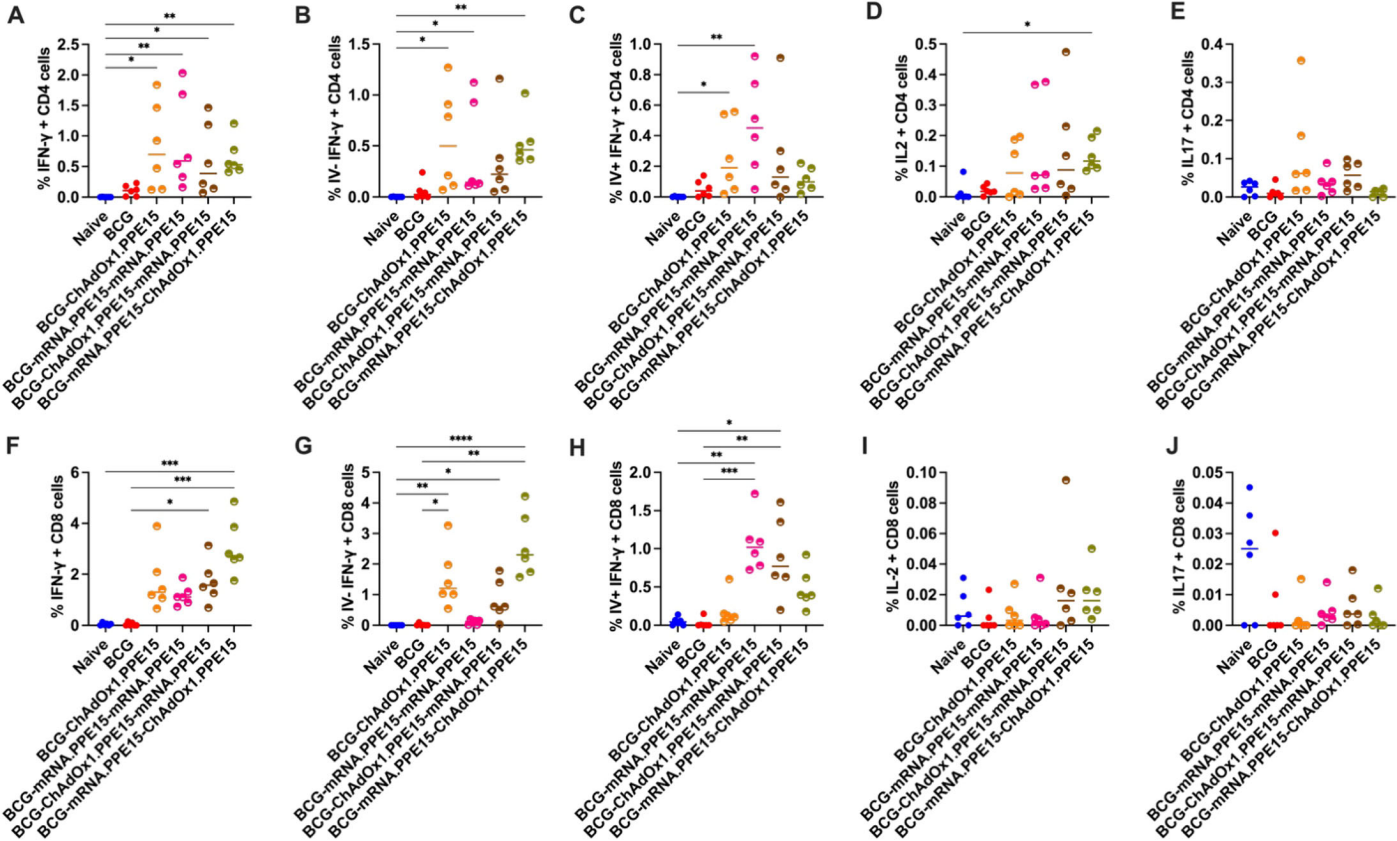
Lung PPE15-specific T-cell responses following heterologous and homologous vaccination as a boost to BCG. Four weeks after the last vaccination lung cells were harvested following intravascular staining with αCD45 and stimulated with PPE15 peptide pool. Flow cytometric analysis of **(A)** IFN-γ, number of CD4+ T cells expressing IFN-γ, **(B)** intravascularly protected, **(C)** intravascularly stained, **(D)** IL-2, and **(E)** IL-17 expression by CD4+ T cells in the lung, in response to stimulation with immunodominant region of PPE15. Panels **(F–J)**, represent the same cytokines but for CD8+ T cells. Each symbol represents response from 1 animal, n=6 per group. The lines indicate the median value for each group. Statistical analyses were performed using Kruskal-Wallis, followed by Dunn’s multi-comparison test to evaluate differences between groups (all pairwise). *p<0.05, **p<0.01, ***p<0.001, ****p<0.0001.

In the lung, IFN-γ CD4+ T cell responses were comparable across all vaccination regimens, except BCG and were significantly higher compared to the naïve group ((N vs B-C (p=0.01), N vs B-M-M (p=0.003), N vs B-C-M (p=0.047), N vs B-M-C (p=0.007), **Figure 6A**). When analysed by compartment, IV- IFN-γ responses were significantly increased in all regimens except BCG-ChAdOx1.PPE15-mRNA.PPE15, with a trend toward higher frequency in the BCG-ChAdOx1.PPE15-mRNA.PPE15 group (N vs B-C (p=0.01), N vs B-M-M (p=0.04), N vs B-C-M (p=0.07), N vs B-M-C (p=0.0019)) (**Figure 6B**). In the intravascular compartment, both BCG-ChAdOx1.PPE15 and BCG-mRNA.PPE15-mRNA.PPE15 regimens induced significantly higher IFN-γ CD4+ T cells compared to naïve control (N vs B-C (p=0.03), N vs B-M-M (p=0.0014), **Figure 6C**). The BCG-mRNA.PPE15-ChAdOx1.PPE15 regimen also induced significant IL-2 responses (N vs B-M-C (p=0.02), **Figure 6D**), while IL-17 responses remained low across all groups (**Figure 6E**).

For CD8+ T cells, all regimens except BCG, induced IFN-γ+ responses, with BCG-mRNA.PPE15-ChAdOx1.PPE15 having significantly higher number than both naïve and BCG-only groups (N vs B-M-C (p=0.0008), B vs B-M-C (p=0.0004), **Figure 6F**). In the parenchymal compartment, BCG-ChAdOx1.PPE15 and BCG-heterologous regimens induced significantly higher IFN-γ responses compared to naïve control (N vs B-C (p=0.003), N vs B-C-M (p=0.0401), N vs B-M-C (p<0.0001, **Figure 6G**). In the intravascular compartment, BCG-mRNA.PPE15-mRNA.PPE15 and BCG-mRNA.PPE15-ChAdOx1.PPE15 induced a significantly higher frequency of IFNγ+ cells compared to naïve control (N vs B-M-M (p=0.002), N vs B-C-M (p=0.018), **Figure 6H**). Among the heterologous vaccination regimens, BCG-mRNA.PPE15-ChAdOx1.PPE15 showed a predominance of IFN-γ+ CD4+ and CD8+ T cells within the parenchymal compartment. In contrast, BCG-ChAdOx1.PPE15-mRNA.PPE15 induced a more balanced distribution of IFN-γ+ T cells across both parenchymal and intravascular compartments. IL-2 and IL-17 responses were low across all regimens (**Figures 6I, J**).

Following stimulation with PPD-T, there were no detectable CD4+ T cell or CD8+ T cell responses in the lung (**Supplementary Figures 3I–N**).

### 3.7 BCG-mRNA.PPE15-ChAdOx1.PPE15 vaccination enriches PPE15-specific lung-resident T cells phenotype in the lung parenchyma

To further characterise the phenotype of the cells in the lung parenchyma and vascular compartments, we utilised intravascular staining in combination PPE15 I-A(b) and H-2D(b) tetramers (Tet).

**Figure 7:**
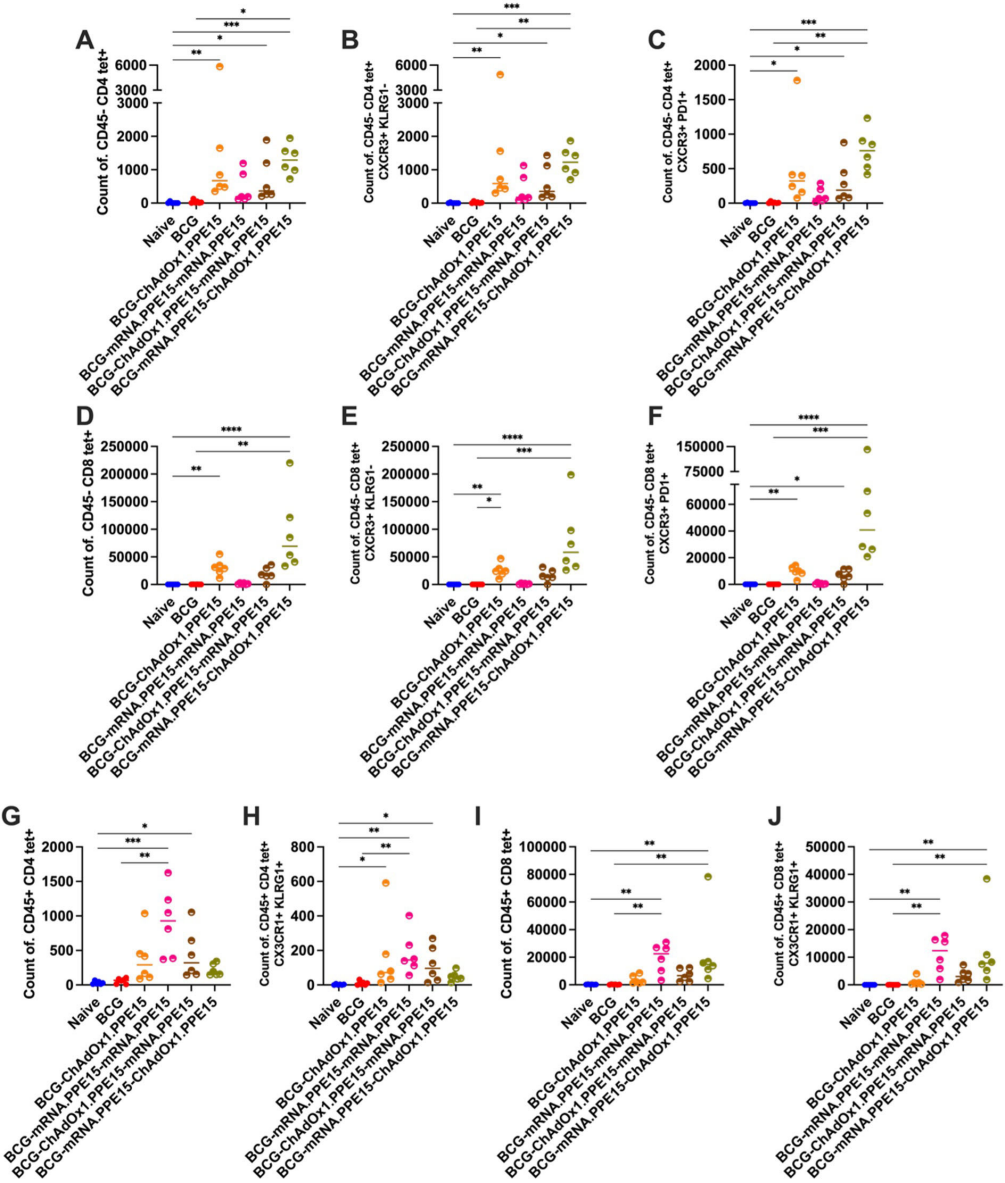
Phenotype characterisation of PPE15-specific T cells in the lung parenchyma and vasculature following heterologous vaccination as a boost to BCG. Four weeks after the last vaccination, PPE15 I-A(b) and H-2D(b) tetramers were combined with intravascular staining with αCD45. Number of tetramer-positive and intravascular staining-negative CD4+ T cells **(A)** that were **(B)** CXCR3+ and KLRG1- **(C)** CXCR3+ and PD1 +. Number of tetramer-positive and intravascular staining-negative CD8+ T cells **(D)** that were **(E)** CXCR3+ and KLRG1- **(F)** CXCR3+ and PD1 +. Number of tetramer-positive and intravascular staining-positive **(G, H)** CD4 + and **(I, J)** CD8 + T cells that were CX3CR1+ KLRG1 +. Each symbol represents response from 1 animal, n=6 per group. The lines indicate the median value for each group. Statistical analyses were performed using Kruskal-Wallis, followed by Dunn’s multi-comparison test to evaluate differences between groups (all pairwise). *p<0.05, **p<0.01, ***p<0.001, ****p<0.0001.

The BCG-mRNA.PPE15-ChAdOx1.PPE15 regimen induced the highest number of PPE15-specific CD4+ T cells in the lung parenchyma, with significantly higher responses compared to the BCG and naïve control groups (N vs B-M-C (p=0.0005), B vs B-M-C (p=0.01). Both BCG-ChAdOx1.PPE15 and BCG-ChAdOx1.PPE15-mRNA.PPE15 also induced antigen-specific T cells in the parenchyma although in lower numbers (N vs B-C (p=0.004), N vs B-C-M (p=0.02). In contrast, BCG-mRNA.PPE15-mRNA.PPE15 did not induce any responses in this compartment (**Figure 7A**). This significance was maintained for cells expressing CXCR3+ KLRG1-, which account for approximately 90% of the CD4Tet+ population, and for cells expressing CXCR3+ PD1+ (N vs B-C (p=0.004,0.01), N vs B-C-M (p=0.03, 0.03), N vs B-M-C (p=0.0004, 0.0002), **Figures 7B, C**).

For CD8Tet+ T cells in the lung parenchyma, significantly higher numbers were observed in the BCG-ChAdOx1.PPE15 and BCG-mRNA.PPE15-ChAdOx1.PPE15 groups compared to naïve control (N vs B-C (p=0.007), N vs B-M-C (p<0.0001), **Figure 7D**). Similar significance was observed for cells expressing CXCR3+ KLRG1-, which account for approximately 90% of the CD8Tet+ population, and CXCR3+ PD1+ expressing cells (N vs B-C (p=0.008, 0.006), (N vs B-M-C (p<0.0001, <0.0001), **Figures 7E, F**). The BCG-mRNA.PPE15-ChAdOx1.PPE15 group had the highest number of CD8Tet+ T cells compared to other regimens.

In the intravascular compartment, BCG-ChAdOx1.PPE15, BCG-mRNA.PPE15-mRNA.PPE15 and BCG-ChAdOx1.PPE15-mRNA.PPE15 induced significantly higher numbers of CD4Tet+ T cells, co-expressing CX3CR1+ and KLRG1+ (N vs B-C (p=0.02), N vs B-M-M (p=0.001), N vs B-C-M (p=0.02), **Figures 7G, H**). In contrast, for CD8Tet+ T cells, significant increases compared to naïve control were observed in the BCG-mRNA.PPE15-mRNA.PPE15 and BCG-mRNA.PPE15-ChAdOx1.PPE15 groups, with these cells also expressing CX3CR1 and KLRG1 (N vs B-M-M (p=0.001), N vs B-M-C (p=0.002) **Figures 7I, J**).

### 3.8 BCG-mRNA.PPE15-ChAdOx1.PPE15 significantly improves BCG efficacy

**Figure 8:**
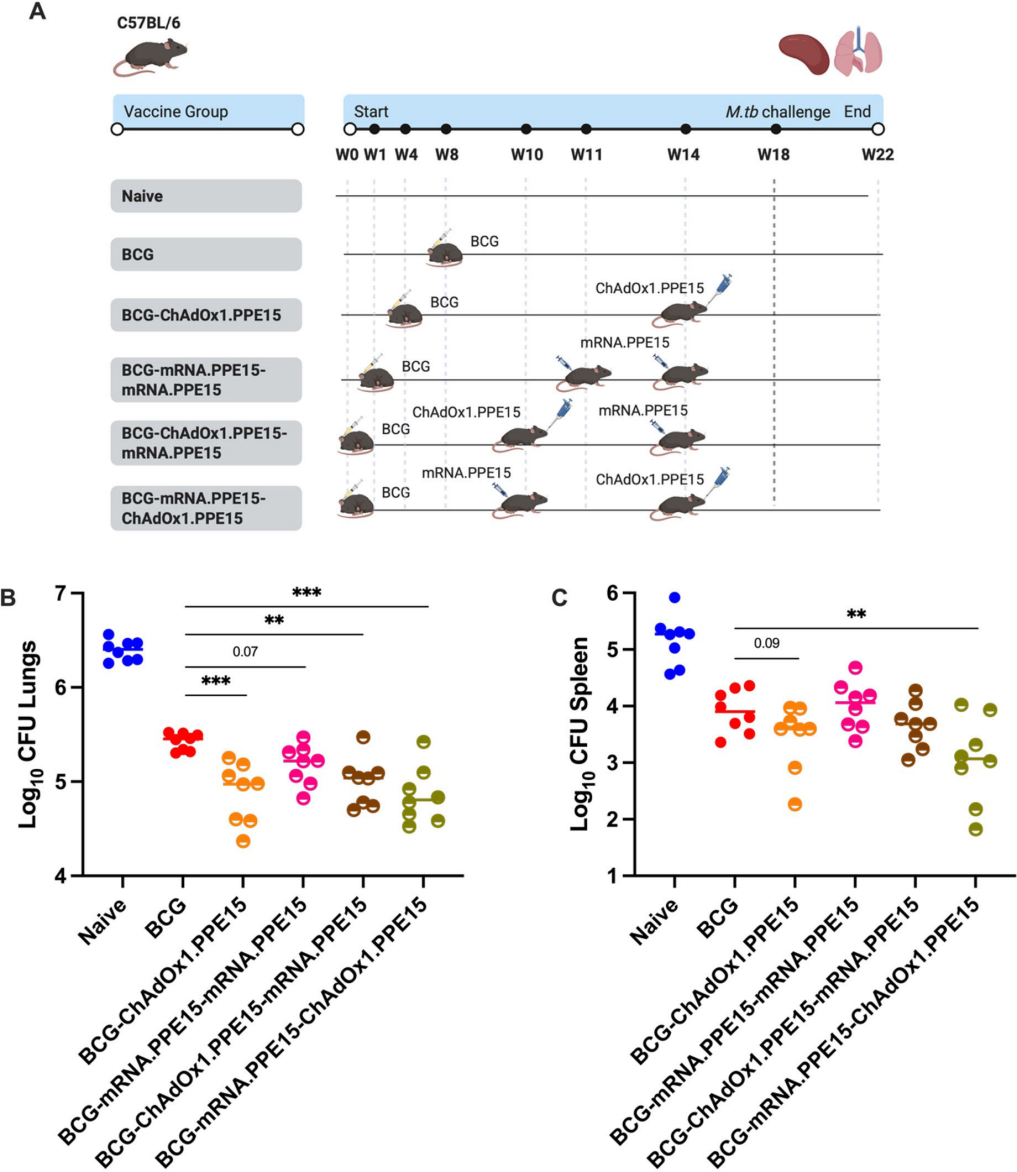
Protective efficacy of ChAdOx1.PPE15, mRNA.PPE15 as heterologous prime boost in BCG primed mice. **(A)** Experimental schema (created with BioRender.com). **(B)** lungs and **(C)** spleens were harvested at the end of the study for colony-forming unit (CFU) enumeration. Each dot represents one animal, n= 8 per group. The lines indicate the median CFU count. Statistical significance was determined using Kruskal-Wallis test with Dunn’s multiple comparisons test (all groups compared to BCG only). *p ≤ 0.05, **p ≤ 0.01, ***p ≤ 0.001.

To determine which vaccine regimens could improve the efficacy of BCG, mice were vaccinated as previously described (**Figure 5A**) and challenged four weeks after the last immunisation (**Figure 8A**). Lungs and spleen were harvested four weeks post challenge for bacterial quantification.

All regimens except BCG-mRNA.PPE15-mRNA.PPE15 improved the efficacy of BCG in the lungs (B vs B-C (p=0.0004), B vs B-M-M (p=0.07), B vs B-C-M (p=0.0036)). The greatest reduction was observed in the BCG-mRNA.PPE15-ChAdOx1.PPE15 group which reduced the load by 0.6 log10 (95%CI 0.81 to 0.38) compared to the BCG-only group (B vs B-M-C (p=0.0002), **Figure 8B**). A similar pattern was observed in the spleen, where BCG-mRNA.PPE15-ChAdOx1.PPE15 significantly improved BCG protection, also achieving a 0.83 log10 (95%CI 1.516 to 0.25) reduction compared to BCG, with some animals in the group having even lower bacteria counts (B vs B-M-C (p=0.0075), **Figure 8C**). There was a trend for lower CFU in the spleen and lung of BCG-mRNA.PPE15-ChAdOx1.PPE15 compared to BCG-ChAdOx1.PPE15-mRNA.PPE15 but this difference did not reach statistical significance (B-M-C vs B-C-M spleen median difference -0.57 log10 95%CI -1.2 to 0.07 p=0.18, lung: -0.17 log10 95%CI -0.45 to 0.13 p=0.49).

## 4 Discussion

Heterologous vaccination, which uses different antigen delivery platforms for priming and boosting, have been shown to improve immune responses compared to homologous approaches, by leveraging the strengths of each platform (5–10, 17). Our study confirmed that such heterologous regimens induce robust cellular and humoral responses, and superior protection against *M.tb* challenge compared to homologous regimens. Importantly, we also varied the route of administration in line with the properties of each platform. The intranasal route was employed for ChAdOx1, leveraging its natural tropism for the lung and our previous findings demonstrating its superior efficacy via this route (30). In contrast, the mRNA platform was delivered intramuscularly, a route shown to be protective against SARS-CoV2, another respiratory pathogen (38, 39). The integration of heterologous platforms with heterologous routes, resulted in a highly effective “prime-pull” regimen, as seen with mRNA.PPE15-ChAdOx1.PPE15, particularly when used as a booster following BCG priming. These findings underscore the potential of tailored heterologous vaccination strategies to enhance TB vaccine efficacy.

Our results demonstrated that whilst mRNA.PPE15 generated a robust Th1 systemic immune response, it was less effective in inducing lung responses. In contrast, the heterologous combination with ChAdOx1.PPE15 improved lung Th1 responses as well as the induction of lung-resident CD4+ and CD8+ TRM-like cells in the parenchyma. The importance of Th1 responses and localisation of these response are critical for early control of *M.tb* and vaccine mediated protection against TB (40). Here we also showed that the order of administration impacted both the immune response and protection; priming with mRNA.PPE15 followed by ChAdOx1.PPE15 led to a higher number of lung CD4+ and CD8+ TRM-like cells compared to other vaccination regimens, suggesting that this sequence optimises T cell trafficking and retention at the site of infection. These observations align with previous studies demonstrating that the order of heterologous vaccination with homologous systemic delivery route, can substantially affect immune outcomes. For instance, ID93 (Rv2608, Rv3619, Rv3620, and Rv1813) protein adjuvanted with oil-in-water emulsion containing TLR4 agonist (GLA-SE) followed by adenovirus type 5 vector expressing ID93 (Ad5-ID93) generated durable CD4+ and CD8+ T cell responses and protection against *M.tb* challenge six months post-vaccination, whereas the reverse and homologous Ad5-ID93 did not (10). Similarly, in TB, priming with a recombinant protein (rMT1721) plus GLA adjuvant followed by a plasmid DNA (MT1721) boost yielded more robust CD4+ and CD8+ T cell responses than reversing the order, which failed to elicit detectable CD8+ T cells (41).

Boosting BCG efficacy is crucial, particularly in countries with a high TB burden where BCG has been part of the national immunisation programmes for decades (42). BCG’s widespread use is due to its well-established protective effect against TB meningitis and disseminated TB in children (43). Additionally, BCG has been shown to provide non-specific immune benefits, including reduced mortality from unrelated respiratory infections (44). In this study, the mRNA.PPE15-ChAdOx1.PPE15 regimen was protective as a standalone vaccine and, more importantly, enhanced the protective efficacy of BCG. The improvement in PPE15-specific responses may be partly due to the high expression of PPE15 by BCG, which could contribute to an effective antigen priming (30). Notably, this regimen induced the strongest IFN-γ responses in the lung parenchyma, where localised T cell immunity is most effective. Supporting this, Woodworth et al. (45) demonstrated that, prior to challenge, vaccinated mice induced higher numbers of IFN-γ-producing cells in the lung parenchyma, which rapidly expanded during infection compared to the control group, correlating with lower CFU counts in the vaccinated group. The BCG-mRNA.PPE15-ChAdOx1.PPE15 induced the highest levels of cytokine secreting CD8+ T cells in the spleen and lung. CD8+ T cells play a critical role in immunity against *M.tb*, working synergistically with Th1 cells to provide optimal protection (46–48). Furthermore, this regimen generated the highest numbers of lung CD4+ and CD8+ TRM-like cells and achieved the greatest reduction in bacterial burden in both lungs and spleen, even surpassing BCG-ChAdOx1.PPE15 and BCG-ChAdOx1.PPE15-mRNA.PPE15. These findings support the conclusion that the sequence of vaccine administration plays a critical role in shaping and enhancing protective immune responses.

The enhanced lung-specific immunity observed in BCG-primed mice vaccinated with mRNA.PPE15-ChAdOx1.PPE15, compared to other regimens, is likely due to a “prime-pull” effect. In this approach, intradermal BCG acts as a systemic prime, inducing broad immunity and specifically activating PPE15-specific T cells through expression of PPE15 by BCG. Worth noting, that PPE family is large and shares a conserved N-terminal domain (49), so the priming effect observed with BCG may not be solely attributable to PPE15, but could also involve responses to other PPE family members. The subsequent intramuscular mRNA.PPE15 boosts this PPE15-specific systemic responses, while the intranasal ChAdOx1.PPE15 “pull” efficiently recruits and retains antigen-specific T cells within the lung parenchyma. In contrast, the BCG-ChAdOx1.PPE15 regimen, which lacks the mRNA-mediated priming, failed to elicit the same magnitude of systemic and lung-localised responses, highlighting the critical role of mRNA.PPE15 in enhancing the priming phase of the heterologous regimen. BCG priming significantly increased PPE15-specific immune responses in both the spleen and lung, with a marked increase in cytokine-producing CD8+ T cells and a moderate increase in CD4+ T cells. It also led to greater numbers of lung TRM-like cells, promoting localisation of antigen-specific responses to the lung. These effects were antigen specific, as PPD-T responses were comparable across all groups (**Supplementary Figure 3**), confirming that the improved responses in the mRNA.PPE15-ChAdOx1.PPE15 group were against PPE15 rather than a generic mycobacterial reactivity. Similar prime-pull strategies have been successfully applied in other respiratory diseases, such as respiratory syncytial virus (RSV) and influenza (25) and are being explored for TB. For example, systemic administration of AdCh68Ag85A followed by an intranasal boost with Ag85 complex protein increased lung TRM cell numbers and improved protection against *M.tb* compared to AdCh68Ag85A alone (26). Similarly, protein-adjuvanted vaccines combined with intranasally administered viral vectors have been shown to increase lung localised immunity (9). Supporting the importance of T cell migration and retention in the lung, Connor et al. (50) demonstrated that blocking lymphocyte trafficking 1 day prior to BCG vaccination with FTY720 reduced protection against *M.tb*, whereas administering it 1 day prior to challenge, but after 9 week following BCG vaccination, had no effect on BCG induced protection. This suggests that the protective efficacy of BCG is dependent on establishing lung-resident lymphocytes. Incorporating FTY720 in future studies may provide further insights into the mechanisms underlying the prime-pull effect. Together, these data support the immunological benefit of heterologous route vaccination, in driving both systemic and mucosal immunity.

BCG-mRNA.PPE15 did not improve the efficacy of BCG which is consistent with previous findings (17). Interestingly, although this regimen induced robust lung PPE15-specific CD4+ and CD8+ T cell responses, the majority of these were localised in the lung vasculature. These data may explain the lack of efficacy observed in this regimen, as the absence of TRM-like cells in the parenchyma limits the ability to mount an effective response against *M.tb*. The intravascular PPE15-specific cells were dominated by CX3CR1+ KLRG1+ T cells, a phenotype that has been shown to be unable to readily migrate to infected lung tissue (29, 45). Moguche et al. (51) also reported that chronic antigen stimulation during *M.tb* infection promotes KLRG1+ T cells, which are terminally differentiated and functionally exhausted. KLRG1-deficient mice have been shown to be more resistant to *M.tb* infection, with increased numbers of activated CD4+ T cells in the lung (52), further underscoring a detrimental role of KLRG1+ T cells in TB immunity.

This study has some limitations. Stimulation assays were performed using the first half of the PPE15 sequence (1–221 out of 391 amino acids), corresponding to the immunodominant region previously identified (30). As a result, the full repertoire of PPE15-specific T cells responses may not have been captured. To quantify bacterial burden accurately, CFU enumeration was performed using whole-lung and whole-spleen homogenates, given the potential for uneven bacterial distribution within the tissue. Because tetramer analyses were performed on lung cell preparations, correlations between parenchymal tetramer-positive T cells and bacterial load were not possible in the current study. Due to cell yield limitations following lung processing, canonical tissue-resident memory markers such as CD69 and CD103 were not included in the tetramer panels. For this reason, these populations as referred to as “TRM-like” (CXCR3+ PD-1+ KLRG1-) cells. Only female C57BL/6 mice were used, and sex- or strain-specific differences in immune responses cannot be excluded. Future studies will assess whether results are replicated in male mice and alternative genetic backgrounds.

In conclusion, this study provides compelling evidence that heterologous vaccination with mRNA.PPE15-ChAdOx1.PPE15, elicits robust cellular and humoral immunity and provides superior protection against *M.tb* compared to homologous regimens. The “prime-pull” effect, where mRNA.PPE15 primes systemic immunity and ChAdOx1.PPE15 pulls these responses to the lung, likely underlies the enhanced efficacy of this regimen. The improved responses in BCG-primed mice further underscore the potential of this strategy as a booster to BCG. Future studies will evaluate the durability of protection and the longevity of lung-resident T cell responses following prime–pull vaccination. In addition, the contribution of innate responses to protection will be investigated. Further optimisation will determine optimal prime–boost intervals, and combinations with additional antigens. Translation of this strategy to human studies will require careful evaluation of the feasibility, safety, and immunogenicity of intramuscular mRNA-LNP vaccination followed by mucosal adenoviral boosting, following. Recent studies have demonstrated that aerosol ChAdOx1 is well-tolerated and capable of inducing both lung mucosal and systemic immune responses (53), supporting its potential for use in prime-pull TB vaccination strategies.

## Data Availability

The raw data supporting the conclusions of this article will be made available by the authors, without undue reservation.
