# Supplementary material for: Enhancing tuberculosis vaccine efficacy with a heterologous mRNA-ChAdOx1 prime-pull strategy targeting lung-resident memory T cells
Source: Front Immunol. 2026 Apr 20;17:1807190. doi: 10.3389/fimmu.2026.1807190 (PMC13136109; doi:10.3389/fimmu.2026.1807190)
Supplement: Supplementary file 1 [file DataSheet1.pdf]

Supplementary Information

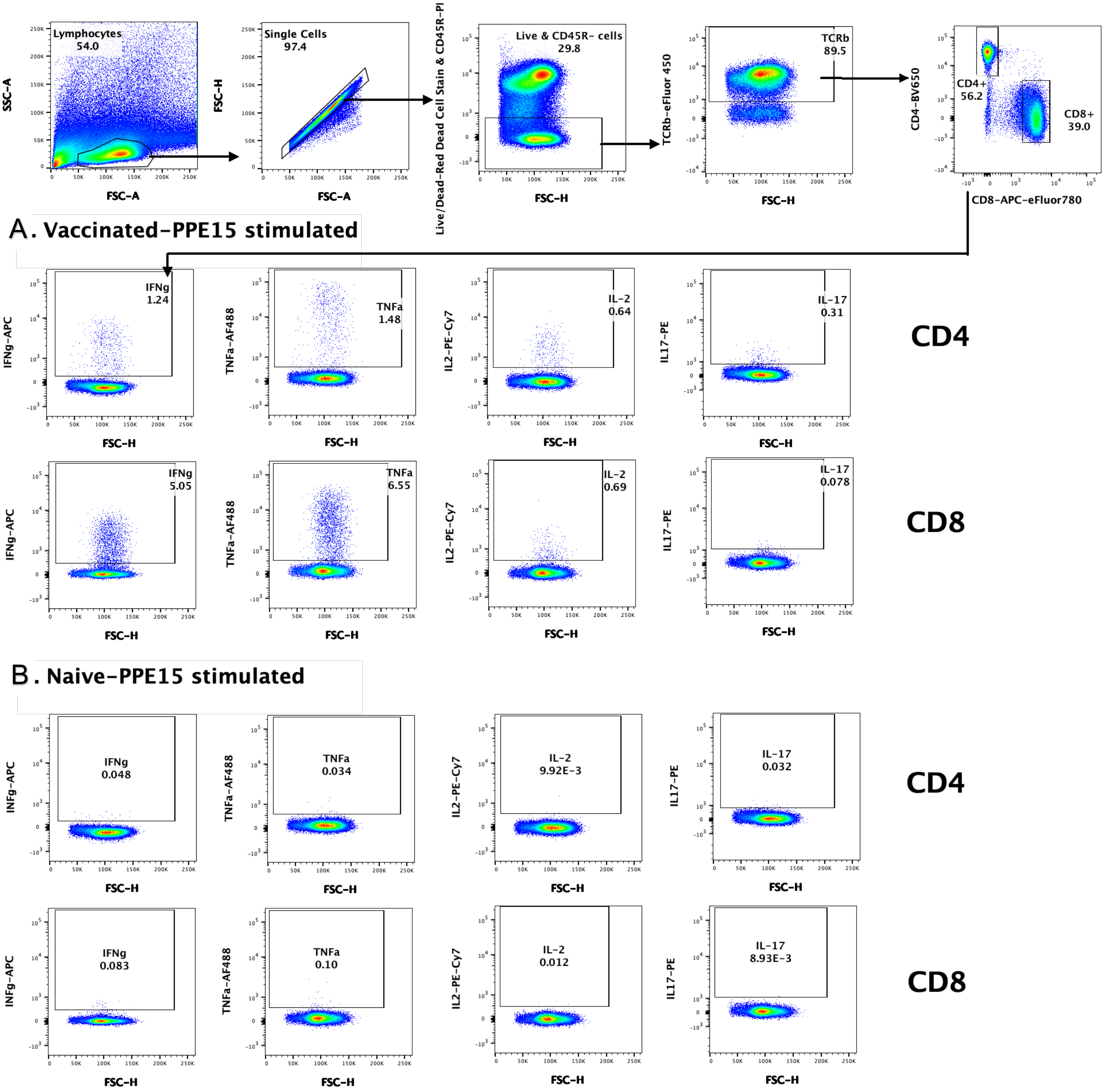

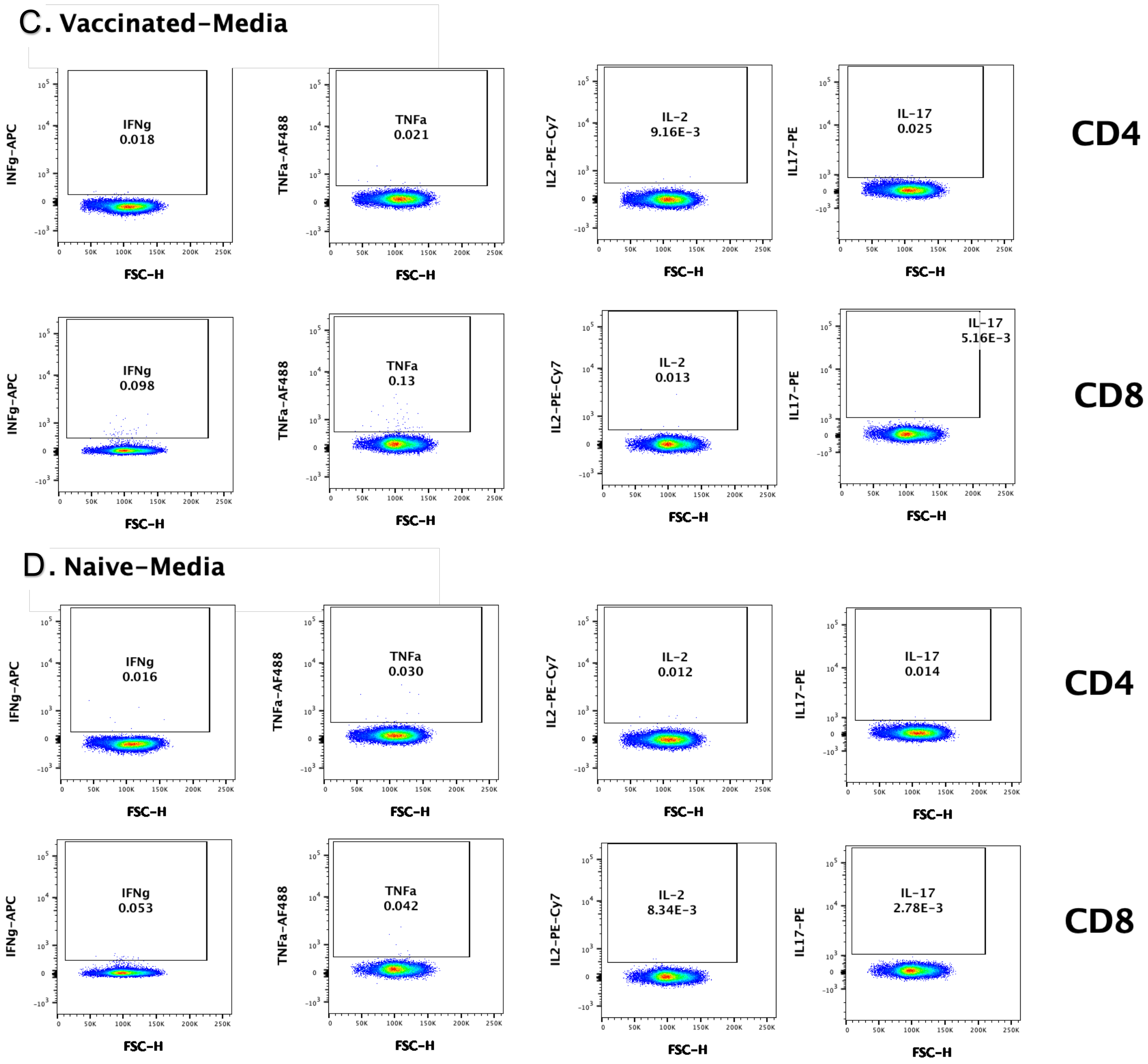

Supplementary Figure 1: Flow cytometry gating strategy for T cell surface and intracellular cytokine staining. Splenocytes from (**A**) vaccinated and (**B**) control animals were stimulated with PPE15 peptide pool to assess cytokine production. Background subtraction was performed using splenocytes from the same animals incubated with media (**C**, **D**). Lymphocytes were gated based on forward scatter (FSC) and side scatter (SSC). Following the exclusion of doublets, live CD45R- cells were selected. TCRβ+ cells were further gated into CD4+ or CD8+ subsets. Cytokine production, including IFN-γ, TNF-α, IL-2, and IL-17, was assessed for each of the CD4+ and CD8+ T cell populations.

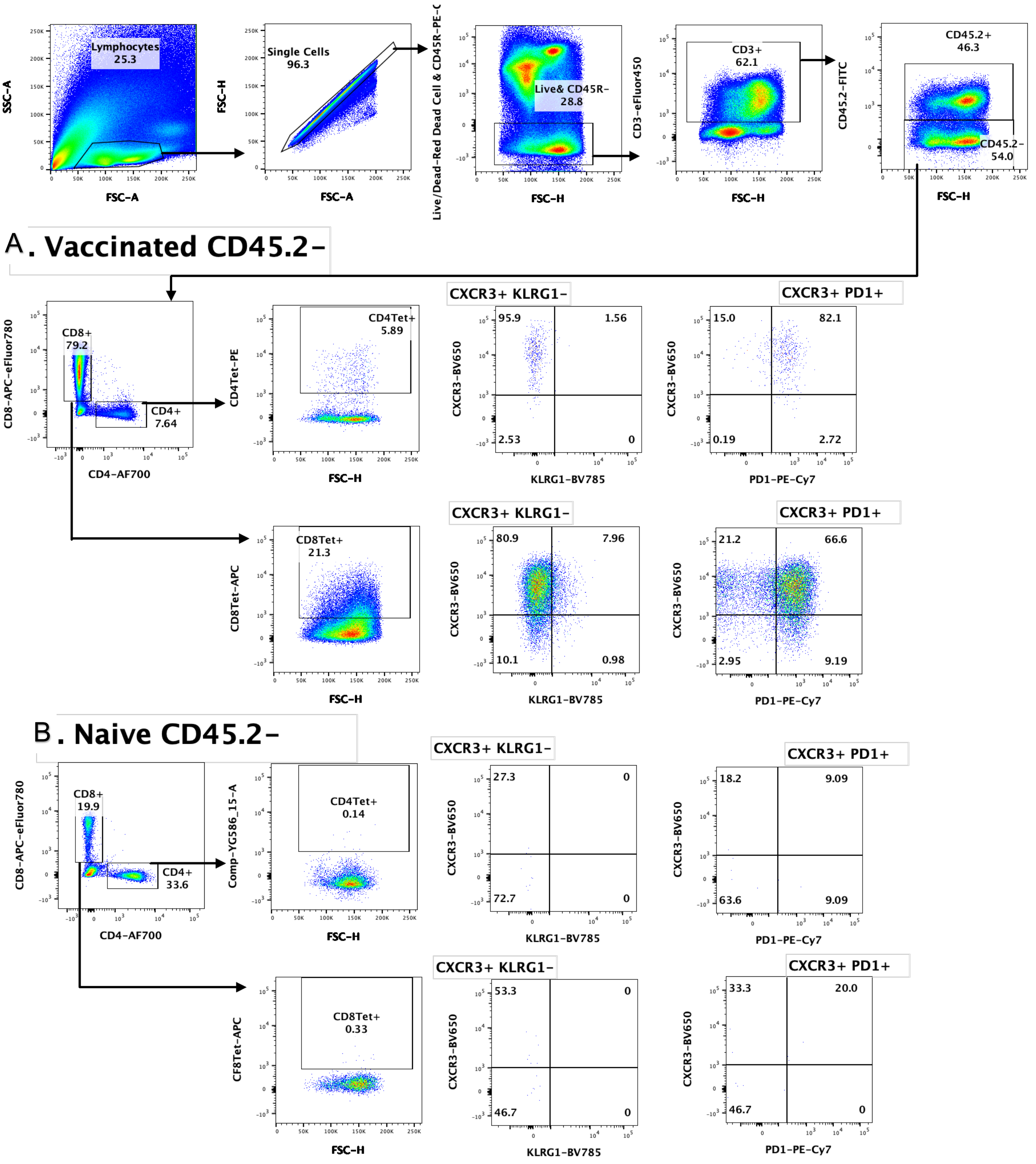
Lymphocytes 25.3
SSC-A
FSC-A
Single Cells 96.3
FSC-H
Live/Dead-Red Dead Cell & CD45R-PE-C
Live& CD45R- 28.8
CD3-eFluor450
CD3+ 62.1
CD45.2-FITC
CD45.2+ 46.3
CD45.2- 54.0
A. Vaccinated CD45.2-
CD8-APC-eFluor780
CD8+ 79.2
CD4+ 7.64
CD4-AF700
CD4Tet-PE
CD4Tet+ 5.89
CXCR3+ KLRG1-
CXCR3-BV650
KLRG1-BV785
95.9
1.56
2.53
0
CXCR3+ PD1+
PD1-PE-Cy7
15.0
82.1
0.19
2.72
CD8Tet-APC
CD8Tet+ 21.3
80.9
7.96
10.1
0.98
21.2
66.6
2.95
9.19
B. Naive CD45.2-
CD8+ 19.9
CD4+ 33.6
Comp-YG586_15-A
CD4Tet+ 0.14
27.3
0
72.7
0
18.2
9.09
63.6
9.09
CF8Tet-APC
CD8Tet+ 0.33
53.3
0
46.7
0
33.3
20.0
46.7
0

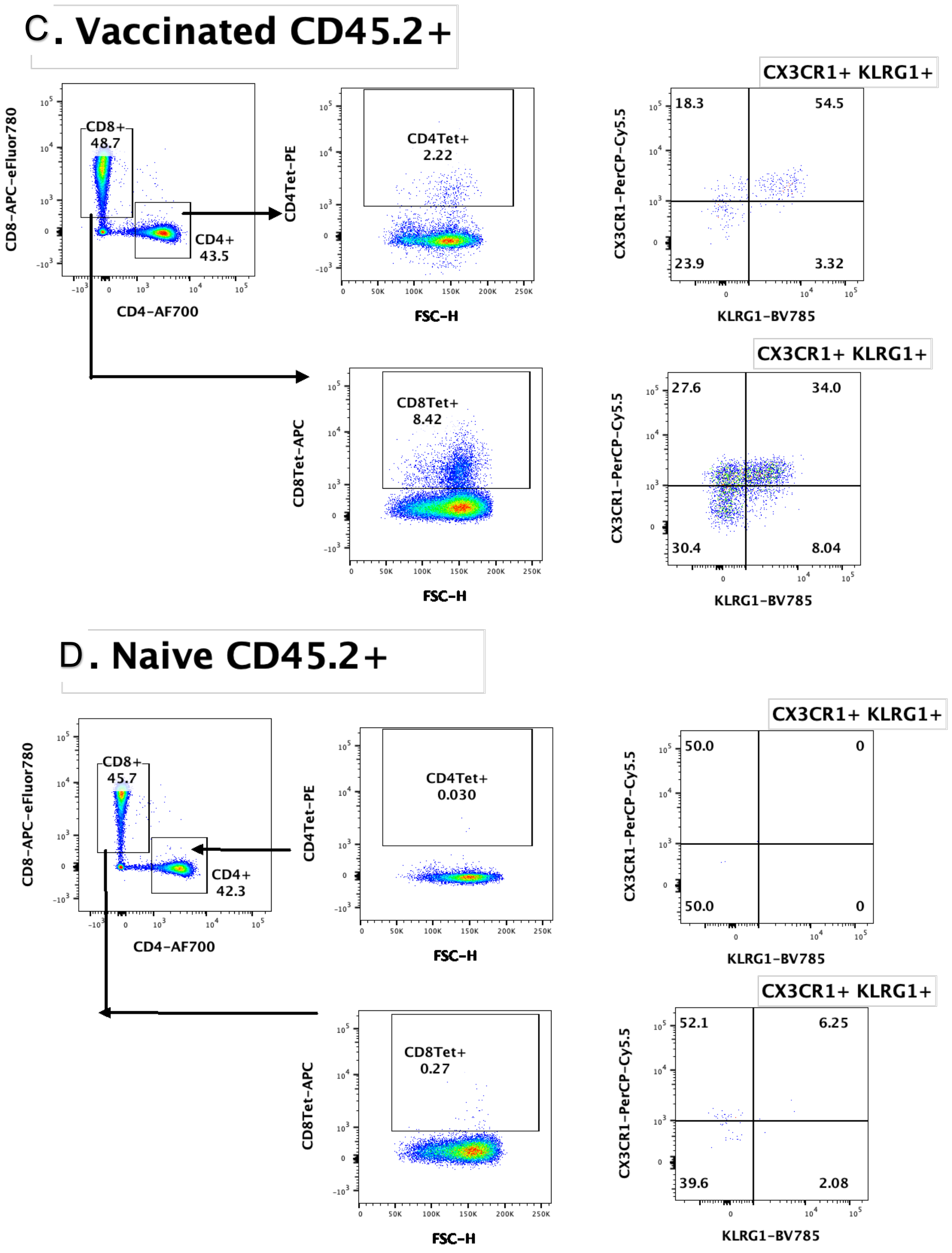

Supplementary Figure 2: Flow cytometry gating strategy for lung resident memory T cells. PPE15 I-A(b) and H-2D(b) tetramers were used in combination with intravascular staining with αCD45. Lymphocytes were gated based on forward scatter (FSC) and side scatter (SSC). After the exclusion of doublets, live CD45R- cells were selected. $CD3^{+}$ cells were subsequently gated into (A, B) CD45.2- (lung parenchymal) and (C, D) CD45.2+ (intravascular) populations. These populations were then gated for CD4+ and CD8+ T cells, followed by further gating for CD4+ PPE15 I-A(b) and CD8+ H-2D(b) tetramer-positive cells. CD45.2-, tetramer-positive cells were analysed for resident memory markers by gating on CXCR3+KLRG1- or CXCR3+PD1+ populations. CD45.2+, tetramer positive CD4+ and CD8+ T cells were gated on CX3CR1+KLRG1+. Representative plots shown from lung cells from vaccinated (A, C) and from (B, D) naïve animals.

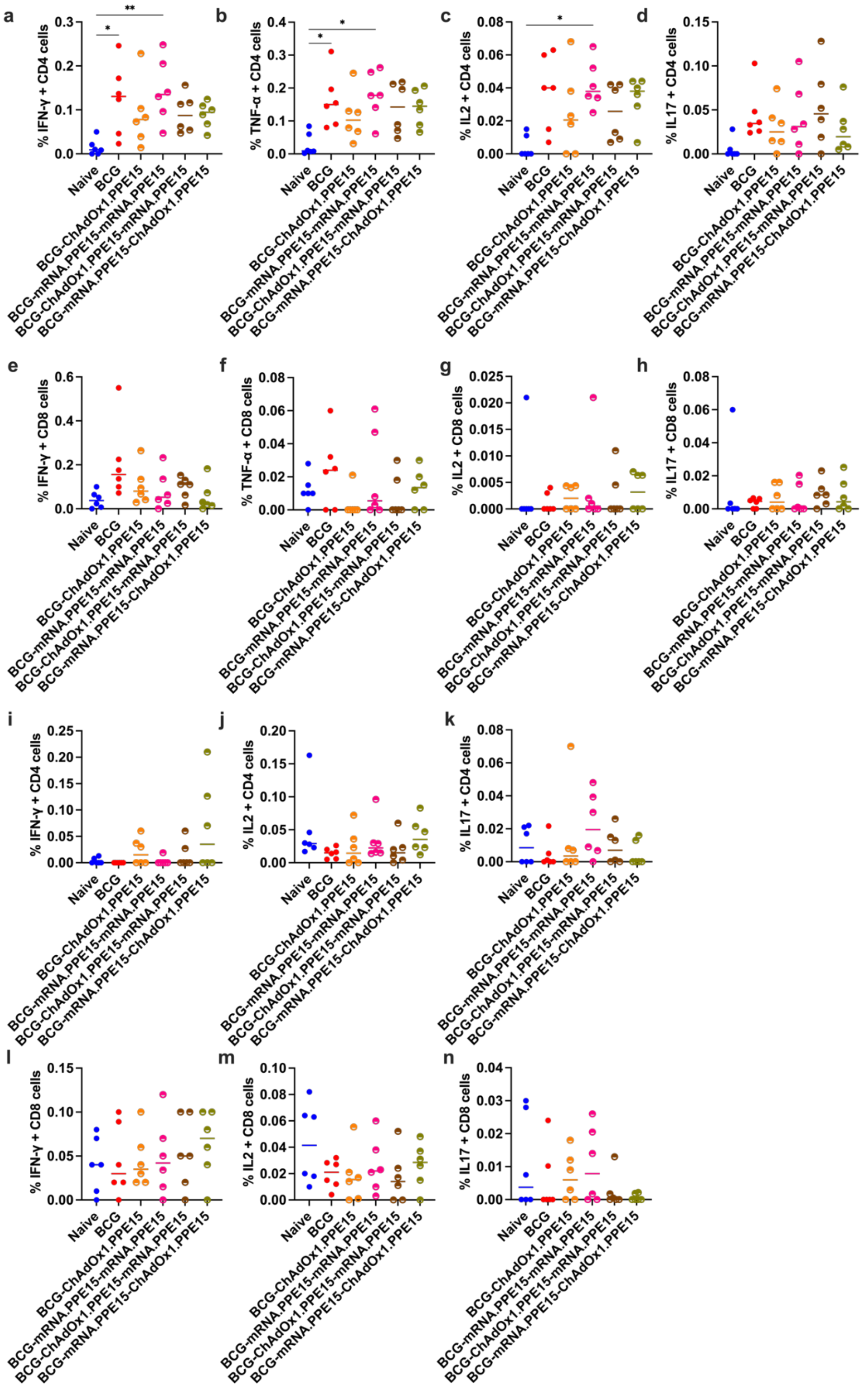

Supplementary Figure 3: Comparable spleen and lung cell responses across vaccinated groups following stimulation with PPD-T. Immune responses were quantified in the lung and spleen four weeks after the last vaccination following cells stimulation with PPD-T. Flow cytometric analysis of spleen (a-d) CD4+, (e-h) CD8+ T cell producing IFN-γ, TNF-α, IL-2, and IL-17. Responses from lung (i-k) CD4+, (l-n) CD8+ T cells producing IFN-γ, IL-2, and IL-17. Each symbol represents response from 1 animal, n=6 per group. The bar indicates the median value for each group. Statistical analyses were performed using Kruskal-Wallis, followed by Dunn's multi-comparison test to evaluate differences between groups. *p<0.05, **p<0.01
